# Biological Effects of New Hydraulic Materials on Human Periodontal Ligament Stem Cells

**DOI:** 10.3390/jcm8081216

**Published:** 2019-08-14

**Authors:** Sergio López-García, Adrián Lozano, David García-Bernal, Leopoldo Forner, Carmen Llena, Julia Guerrero-Gironés, José M. Moraleda, Laura Murcia, Francisco J. Rodríguez-Lozano

**Affiliations:** 1 Cellular Therapy and Hematopoietic Transplant Unit, Internal Medicine Department, Virgen de la Arrixaca Clinical University Hospital, IMIB-Arrixaca, Campus Regional de Excelencia Internacional “Campus Mare Nostrum”, University of Murcia, 30120 Murcia, Spain; 2 Department of Genetics and Microbiology, Faculty of Biology, University of Murcia, 30100 Murcia, Spain; 3 Department of Stomatology, University de Valencia, 46010 Valencia, Spain; 4 Department of Stomatology, University of Murcia, 30008 Murcia, Spain

**Keywords:** hydraulic cements, bioceramics, cytotoxicity, endodontic cements, human periodontal ligament stem cells

## Abstract

*Background*: The aim of this study was: to evaluate the biological properties of new hydraulic materials: Bio-C Repair and Bio-C Sealer. *Methods*: Periodontal ligament stem cells were exposed to several dilutions of Bio-C Repair and Bio-C Sealer. The ion release profile and pH were determined. Metabolic activity, cell migration and cell survival were assessed using the 3-(4, 5-dimethylthiazol-2-yl)-2, 5-diphenyltetrazolium bromide (MTT), wound-healing assays and Annexin assays, respectively. Cells were cultured in direct contact with the surface of each material. These were then analyzed via scanning electron microscopy (SEM) and energy dispersive X-ray (EDX). Statistical differences were assessed using a two-way ANOVA (*α* < 0.05). *Results*: Similar pH was observed in these cements. Bio-C Sealer released significantly more Ca and Si ions (*p* < 0.05) in comparison with Bio-C Repair. Undiluted Bio-C Sealer induced a significant reduction on cellular viability, cell survival and cell migration when compared to the control (*p* < 0.05). Moreover, SEM showed abundant cells adhered on Bio-C Repair and a moderate number of cells attached on Bio-C Sealer. Finally, EDX analysis identified higher percentages of Ca and O in the case of Bio-C repair than with Bio-C sealer, while other elements such as Zr and Si were more abundant in Bio-C sealer. *Conclusions*: Bio-C Repair displayed higher cell viability, cell adhesion and migration rates than Bio-C Sealer.

## 1. Introduction

Hydraulic materials, also known as hydraulic silicate-based materials or bioactive cements (BECs), are new materials which leach ions, with potential influence on reparative/regenerative responses [1]. These new materials represent an improvement over mineral trioxide aggregate MTA-based materials by minimizing drawbacks such as tooth discoloration, relatively difficult handling, long setting times or the release of heavy metal elements [2,3,4]. 

The materials have been proposed for direct pulp capping, pulpotomy or apexification [5,6]. However, it should be pointed out that during perforation repair, vital pulp therapy and retrograde filling, the cytotoxicity of the material used can inhibit or alter the growth of various cell types, inhibiting tissue repair [7].

Recently, two new materials Bio-C Repair (repair cement) and Bio-C Sealer (endodontic sealer) (Angelus, Londrina, PR, Brazil) were placed on the market in the form of premixed bioceramic materials with the same biological interactions as mineral trioxide aggregate but providing improvements in terms of manipulation and insertion. These materials contain calcium silicates, which are hydrated by contact with the local humidity, producing a hydrated calcium silicate structure and calcium and hydroxyl ions, incorporating zirconium oxide as a radiopacifier [8]. 

Human periodontal ligament stem cells (hPDLSCs) have emerged as a promising cell model for cytotoxicity studies involving endodontic sealers because these cells may be in direct contact with unintentional sealer extrusions [9]. hPDLSCs are cells with the multidirectional ability to differentiate into cementoblast-like cells, adipocytes, and collagen-forming cells. [10]. Hence, hPDLSCs were considered to be potential seed cells in cytotoxic studies with endodontic cements [11,12]. 

Cytotoxicity tests with cells allow us to understand the biological effects caused by a material or its extract in cell culture, since it provides results that are suitable for quantitative assessment [13]. The International Organization for Standardization (ISO) 10993-5 guideline recommends three types of cytotoxicity test methods: indirect contact, direct contact, and extract dilution tests [14]. To date, there have been no published studies evaluating how these new materials could affect the cell proliferation or cell attachment to these materials.

Considering this background information, the present study was designed to evaluate the cytotoxicity of Bio-C Sealer and Bio-C Repair in periodontal cells. The null hypothesis of the current study is that there is no significant difference between these biomaterials in regard to cytotoxic and apoptotic effects.

## 2. Material and Methods

### 2.1. Material Extracts

BECs tested in this study were Bio-C Repair and Bio-C Sealer (Angelus, Londrina, Brazil). The studied BECs chemical compositions, supplied by the manufacturers, are listed in Table 1.

The material discs were molded in a sterile cylindrical polyethylene tube (diameter: 5 mm; height: 2 mm), kept in an incubator at 37 °C for 24 h to achieve complete setting, and then sterilized by ultraviolet irradiation for 1 h as reported previously [15]. Next, eluate extraction was performed in sterile conditions using Dulbecco’s Modified Eagle Medium (DMEM) culture medium as the extraction vehicle. The obtainment procedure was as follows: the materials were stored in the culture medium for 24 h at 37 °C in a humid atmosphere containing 5% CO_2_. This procedure was carried out according to International Organization for Standardization (ISO) guideline 10993-12 and the ratio of material surface area and extraction vehicle volume was calculated as 1.5 cm^2^/mL. The extraction medium was filtered with a 0.22 µm filter. Undiluted extract media was reserved for experimental procedures and two dilutions of 1/2 and 1/4 in DMEM media were prepared.

### 2.2. Ion Release Analysis and pH

Three samples of the studied cement types measuring 5 mm in diameter and 2 mm high were prepared in 5 mL milli-Q water, and the presence of calcium, silicon, strontium, and zirconium was assessed using inductively coupled plasma-mass spectrometry (ICP-MS Agilent 7900, Stockport, UK). The pH of the different extracts was determined using a twin pH meter (GLP21+, Crison, Barcelona, Spain). Results are represented as the mean ± standard deviation.

### 2.3. Isolation and Culture of hPDLSCs

The process of cell isolation and culture was managed as previously described [16]. This study followed the ethical precepts of the Declaration of Helsinki and was approved by the local ethics committee (University of Murcia; Ref 7365-12). hPDLSCs were carefully isolated from the root by enzyme digestion method and cultured in α minimum essential medium (α-MEM, Gibco, Life Technologies, California, CA, USA) supplemented with 10% fetal bovine serum (FBS, Gibco, Life Technologies, California, CA, USA), 100 μL/mL streptomycin, and 100 U/mL penicillin in an incubator at 37 °C in 5% CO2. Cells at 80% confluence were subcultured by 3 mg/mL trypsin (Gibco, Life Technologies, California, CA, USA) at a ratio of 1:3. hPDLSCs at the third passage were used for the following experiments.

### 2.4. Expression of Mesenchymal Stem Cell Surface Markers

The immunophenotype of hPDLSCs was evaluated by flow cytometry after the third passage, and the expression of mesenchymal stem cell surface markers was analyzed using flow cytometry. Briefly, hPDLSCs were washed with phosphate buffered saline (PBS) and incubated in the dark for 15 min at 4 °C with the following antibodies: APC-conjugated anti-CD73 (clone AD2), FITC-conjugated anti-CD90 (clone DG3), PE-conjugated anti-CD105 (clone 43A4E1), and PerCP-conjugated anti-CD34 (clone AC136), CD45 (clone 5B1), CD14 (clone (TÜK4), and CD20 (clone LT20.B4) (all from Miltenyi Biotec, Bergisch Gladbach, Germany). After labeling, cells were washed twice, resuspended in PBS, and analyzed in a FACSCalibur flow cytometer (at least 50,000 cells per condition). The results were analyzed using FlowJo software (FlowJo LLC, Ashland, OR, USA) [17]. The medium was refreshed every 3 days. Also, to analyze the in vitro multipotential differentiation ability of hPDLSCs, cells were cultured in OsteoDiff media, AdipoDiff media, and CondroDiff media (Miltenyi Biotec, Bergisch Gladbach, Germany) for 4 weeks to induce osteogenic, adipogenic, and chondrogenic differentiation, respectively. Osteogenesis was demonstrated by mineralization and assessed by Alizarin red staining (Sigma-Aldrich, St. Louis, MO, USA). Adipogenesis was evaluated with Oil Red O solution (Sigma-Aldrich, St. Louis, MO, USA) to detect accumulation of neutral lipid droplets. Finally, chondrogenic differentiation was verified with Alcian blue staining (Sigma-Aldrich, St. Louis, MO, USA) to detect glycosaminoglycans.

### 2.5. Cell Viability Assay

The cytotoxicity of the extracts to the hPDLSCs was assessed using the 3-(4, 5-dimethylthiazol-2-yl)-2, 5-diphenyltetrazolium bromide (MTT) assay. The eluates from three discs of each material were obtained after immersing them in culture medium for 24 h. Briefly, 1 × 104 hPDLSCs were added to 96-well plates with 180 μL of DMEM for 24 h. Then, extracts of the materials were added, and cells incubated at 37 °C in a 5% CO2 for 24, 48 or 72 h. The samples were incubated with 1 mg/mL of MTT for 4 h at the indicated time points. Then, 0.2 mL of dimethyl sulfoxide (DMSO) was added to each well to solubilize the formazan crystals obtained as a result of MTT reduction by the viable cells. The optical density value was measured by spectrophotometer (Synergy H1, BioTek, Winooski, VT, USA) at 570 nm (Abs570). The data obtained by each group were normalized based on cells + medium.

### 2.6. Cell Migration

Cell migration test was performed using a “wound-healing” test (ibidi GmbH, Munich, Germany). The eluates from six discs of each material were obtained after immersing them in culture medium for 24 h. The hPDLSCs were cultured on 12-well plastic plates until they grew to form a monolayer. A crossed area was scratched with a 100-μL pipette tip, and the cell debris was removed with PBS. The healing process was allowed to proceed in the absence (control group) or in the presence of biomaterial eluates. Observation of the “wound area” was performed at 24, 48, and 72 h. The wound closure areas were measured with ImageJ (National Institutes of Health, Bethesda, MD, USA) to calculate the percentage of wound area after 24, 48 or 72 h relative to the total wound area measured at 0 h in the same well. Migration distances were analyzed separately during periods 0–24 h (migration during first 24 h period), 24–48 h (during second 24 h period), and 48–72 h (during third 24 h period). In order to avoid scratch width variation, “relative wound closure” area (RWC) was calculated (RWC [%] = wound closure area [pixel] × 100 [%]/x [pixel]).

### 2.7. Analysis of Apoptosis and Necrosis by Flow Cytometry (Annexin V/7-AAD Staining)

After incubation for 24 h, hPDLSCs were exposed to cement extracts (*n* = 6) at 1:1, 1:2, and 1:4, and the cells were harvested at day 3 by trypsin. Cells were resuspended in the wash buffer and cell concentrations were adjusted to 1 × 10^6^/mL/sample. Then, cells were rinsed with 0.01 mol/L phosphate buffered saline (PBS) twice and resuspended in 100 μL wash buffer. Cell apoptosis rates were analyzed by FAC-Scanflow cytometer (BD Biosciences, San Jose, CA, USA). Percentages of live (Annexin-V−/7-AAD−), early apoptotic (Annexin-V+/7-AAD−) or late apoptotic and necrotic (Annexin-V+/7-AAD+ and Annexin-V-/7-AAD+) cells were determined by flow cytometry. Cells cultured without eluates were used as a negative control. Each condition was analyzed in triplicate.

### 2.8. Scanning Electronic Microscopy and Energy-Dispersive X-Ray Analysis (SEM/EDX)

Six discs of both Bio-C Repair and Bio-C Sealer were prepared as previously described, dispensed on 24-well plates, and subdivided into two groups (three of them were used to analyze the attachment of the cells by SEM). A total of 5 × 10^4^ hPDLSCs were directly added onto each disk and cultured for 72 h. The cells were fixed using 4% glutaraldehyde in PBS for 4 h at 4 °C. Subsequently, the samples were dehydrated in increasing ethanol solutions before critical point drying. Specimens were mounted on aluminum stubs, followed by gold-palladium (Au-Pd) coating (Bio-RAD Polaron e5400 SEM Sputter Coating System, Kennett Square, PA, USA). Finally, the samples were observed with SEM to elucidate the interaction between cells and material (100 and 300×).

Representative surfaces of BECs and elemental analysis were studied as previously described [16]. Scanning electron microscopy (SEM; JSM-635F, JEOL, Tokyo, Japan) equipped with energy dispersive X-ray spectroscopy (EDX) was used to perform this experiment. An accelerating voltage of 20 kV was used to acquire SEM images. EDX provided microchemical spectra and semi-quantitative compositional analysis (weight% and atomic% of the elemental composition).

### 2.9. Statistical Analysis

Data were analyzed using SPSS version 22.0 statistical software (SPSS, Inc., Chicago, IL, USA). Each experiment was performed with three replicates and carried out at least three times. Quantitative data are presented as the mean ± standard deviation (SD). After verifying the homogeneity of variances, cytotoxicity data were analyzed statistically by a two-way ANOVA followed by the Bonferroni correction. A *p-*value < 0.05 was considered significant.

## 3. Results

### 3.1. Ion Release and pH

A similar pH was observed in BCEs immersed in DMEM. Bio-C Sealer released significantly more Ca and Si ions (*p* < 0.05) in comparison with Bio-C Repair. However, the release of Zr was similar in both materials. In addition, the presence of strontium was higher in Bio-C Repair (Table 2).

### 3.2. Isolation and Identification of hPDLSCs

**Figure 1:**
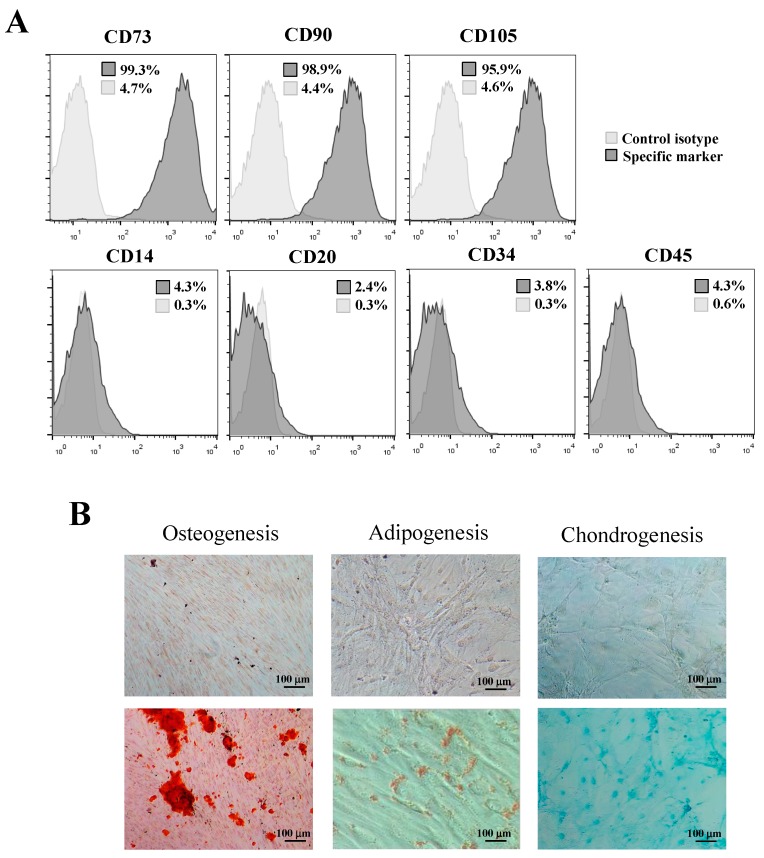
(**A**) Expression of mesenchymal stem cells markers CD73(99.3%), CD90(98.9%), and CD105(95.9%), and hematopoietic markers CD14(4.3%), CD20(2.4%), CD34(3.8%), and CD45(4.3%). Insert numbers inside histograms represent percentages of positive cells for each marker. Control isotypes staining are also shown. (**B**) Osteogenic, adipogenic, and chondrogenic differentiation of hPDLSCs (Alizarin red, Oil red O, and Alcian blue staining, respectively). Negative controls are also shown (top images). Scale bar: 100 μm.

The hPDLSCs were sorted by magnetic-activated cell sorting of generation P3. Cells exhibited a typical fibroblast-like spindle-shaped appearance and had clone-forming ability. Flow cytometry confirmed that they expressed the mesenchymal stem cell surface markers CD73, CD90, and CD105, but did not express the hematopoietic-specific cell markers CD14, CD20, CD34 or CD45 (Figure 1A). Alizarin red, Oil red O, and Alcian blue staining assays confirmed that the hPDLSCs could be induced to differentiate into osteoblasts, adipocytes, and chondrocytes, respectively (Figure 1B).

### 3.3. MTT Assay

**Figure 2:**
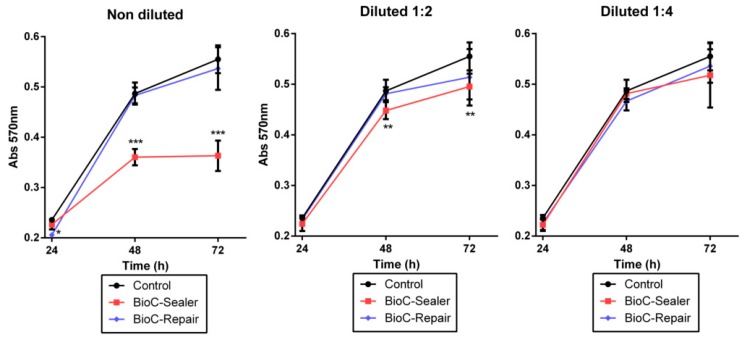
Viability of periodontal ligament stem cells incubated in the presence of different endodontic cement extracts (* *p* < 0.05; ** *p* < 0.01; *** *p* < 0.001, respectively).

The cytotoxicity results of the control and experimental groups are shown in Figure 2. After 72 h of culture, Bio-C Repair exhibited similar cell viability when compared to the control condition with no significant difference between them. Conversely, undiluted and ½ dilution of Bio-C Sealer produced a statistically significant reduction in cellular viability after 72 h (*** *p* < 0.001; ** *p* < 0.01, respectively). However, no significant differences in cell viability were observed after incubation with ¼ dilution of Bio-C Sealer compared to the control group (treated with culture media only) (Figure 2).

### 3.4. Migration Assay

**Figure 3:**
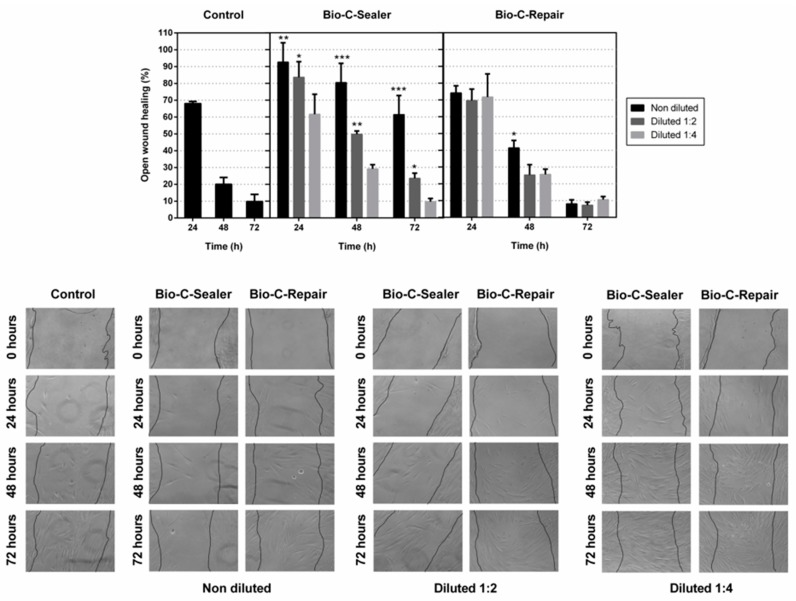
Effect of endodontic cement eluates on cellular migration on a wound healing assay. Confluent hPDLSCs were wounded and incubated with different dilutions of the material extracts for up to 72 hours. Cell migration is represented as the percentage of the open wound area for each condition compared with the control. Significant differences are indicated as * *p* < 0.05; ** *p* < 0.01; *** *p* < 0.001, respectively.

The wound area was determined after the removal of the scarring insert and percentage of open wound was determined in the following 24, 48 and 72 h. hours. Treatment with 1/1 and 1/2 dilutions of Bio-C Sealer induced a significantly lower cell migration rate than observed using complete medium (control) (* *p* < 0.05; ** *p* < 0.01; *** *p* < 0.001) (Figure 3). However, Bio-C Repair promoted wound closure after 72 h of treatment, obtaining comparable levels of migration to those observed with the control group. Collectively, the results showed that Bio-C Repair and 1/4 dilution of Bio-C Sealer allowed the migration of hPDLSCs.

### 3.5. Apoptosis/Necrosis Assay

**Figure 4:**
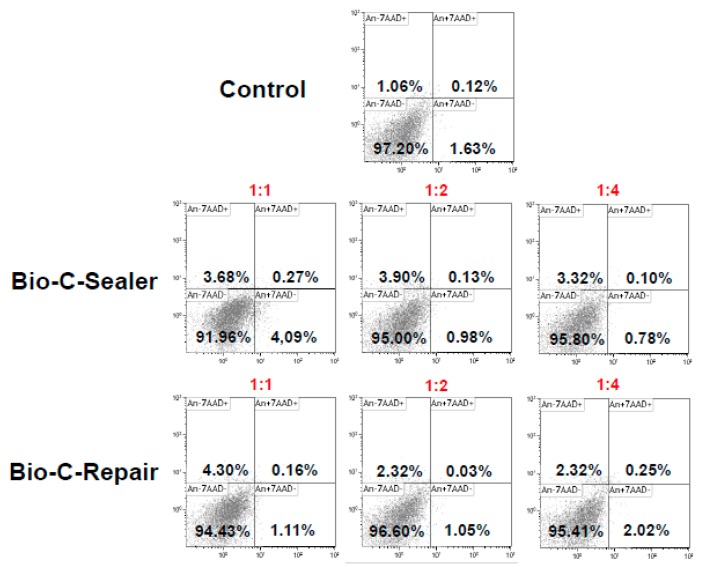
Cell survival in presence of bioactive cements (BECs) was performed by Annexin-V and 7-AAD double-staining. The data represent one of three independent experiments. (i) Lower left quadrant represents the viable cells; (ii) lower right quadrant shows the early apoptosis cells; (iii) upper right quadrant signifies the late apoptosis cells; and (iv) the upper left quadrant indicates dead cells.

Figure 4 shows the results of flow cytometry, used to study the apoptotic/necrotic effect of the BEC eluates on hPDLSCs. The percentages of cell survival with undiluted extracts of Bio-C Sealer and Bio-C Repair were 91.96% and 94.42%, respectively, and with 1:2 and 1:4 dilutions of BECs the percentages of cell survival were ≥95%.

### 3.6. Scanning Electronic Microscopy and Energy-Dispersive X-ray Analysis (SEM/EDX)

**Figure 5:**
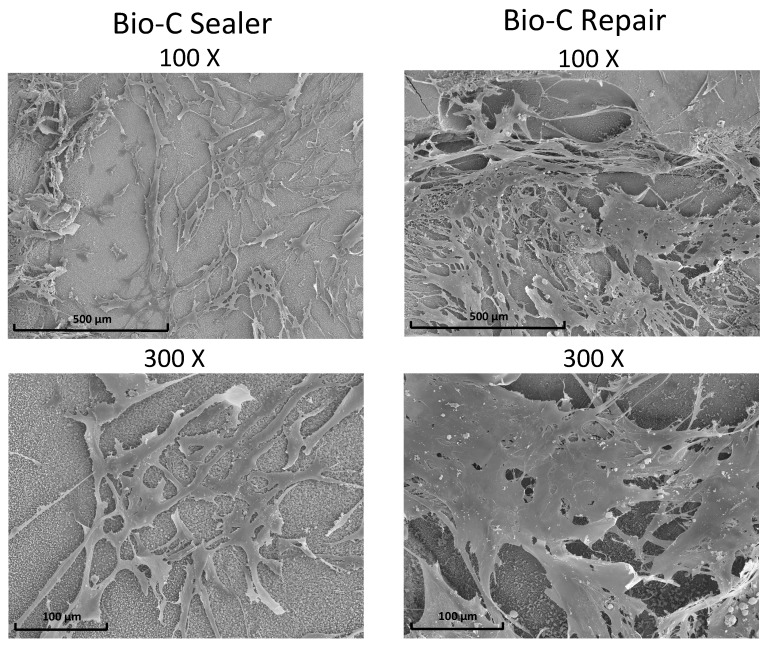
Scanning electron microscopic photomicrographs showing morphological aspects of *h*PDLSCs on discs of Bio-C Sealer and Bio-C Repair at 72 h. Scale bar: 500 μm and 100 μm. Magnification: 100× and 300×.

The morphology and adhesion of hPDLSCs on the surface of Bio-C Sealer and Bio-C Repair after culture for 72 h are shown in Figure 5. After 72 h of culture, cells were well individualized, flattened, and spindle-like in shape, with multiple prolongations in the case of Bio-C Repair. However, in the Bio-C Sealer group, less elongated and spindle-shaped cells were found on the surface (scale bar: 100 μm and 500 μm).

**Figure 6:**
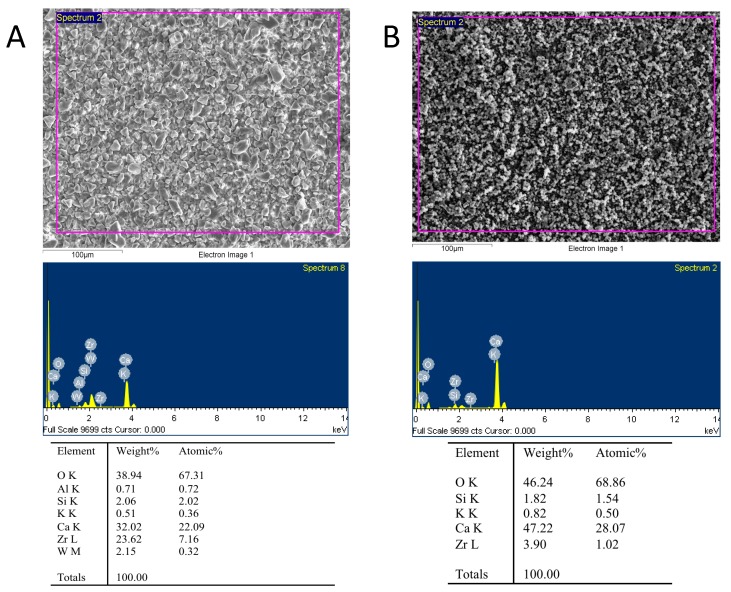
Surface properties and composition of Bio-C Sealer (**A**) and Bio-C Repair (**B**) under scanning electron microscope with energy-dispersive X-ray analysis (SEM-EDX).

EDX was used as an analytic technique for chemical characterization and identification of the major elements present in each disc samples. As displayed in Figure 6, EDX detected the presence of oxygen, silicon, calcium, potassium, and zirconium in Bio-C Sealer or Bio-C Repair with different percentages. No carbon was found in Bio-C Sealer or Bio-C Repair (Figure 6). Traces of aluminum and tungsten were only found in Bio-C Sealer.

## 4. Discussion

Hydraulic materials are composed of tricalcium silicate, dicalcium silicate or tricalcium aluminate and capable of producing hydroxyapatite when incorporated with calcium and silicon, showing functional bonding with dentin [18]. This characteristic makes them suitable for use either as a repair material or as a sealer for root canal fillings [17]. These materials often interact with stem cells from periapical tissues, producing a biological seal and inducing the healing process [15,19]. In fact, an important consideration when selecting resources to be used during cytotoxicity testing is the cells and tissues that are likely to be affected by the tested agent [13]. For these reasons, hPDLSCs were used in the present study.

Several studies have been developed in order to analyze the possible adverse effect of materials in cells, animals, and humans [20,21]. However, novel cell culture methods have been introduced in an attempt to replace animal models [22]. The advantages of using extracts are that they can be sterilized by filtration, facilitating the evaluation of their effects on the cells, and they simulate a clinical situation, in which the substances leach and diffuse into the periapical tissue [23].

By definition, an ideal sealer must have adequate biocompatibility and physicochemical properties, be bioactive, and demonstrate antimicrobial activity [24]. The biocompatibility of the new endodontic materials for clinical use is an important issue to be evaluated. Thus, in this study we evaluated the in vitro cytotoxicity of the new two endodontic cements studied: Bio-C Repair and Bio-C Sealer.

The results of ion release evidenced the release of Ca^2+^ in both materials. However, Ca^2+^ release was more intense in Bio-C Sealer than Bio-C Repair. Calcium silicate cements exhibit the formation of calcium hydroxide during the early stage of the setting process. The release of OH- and Ca^2+^ ions is related to the solubility of the biomaterials and their antimicrobial properties [25,26]. Probably, the difference of liquid/solid ratio in these BECs is correlated with the differences in ion release.

The classic assay to evaluate the possible cytotoxic effects of materials is the MTT assay after 24 h of cell exposure, following ISO 10993 guidelines [27]. The MTT assay revealed that Bio-C Repair had high cell viability rates at all the dilutions tested (undiluted, 1/2, 1/4), whereas significant differences were observed with undiluted and 1/2 dilutions of Bio-C Sealer compared to the control group at the same time points. Previous reports have demonstrated the good cytocompatibility of new bioceramic cements [28,29]. Notably, Vouzara et al. [30] showed that Bioroot RCS, a calcium silicate root canal sealer, was significantly less cytotoxic than MTA Fillapex and SimpliSeal. Other authors have suggested that new bioceramics, such as iRoot BP Plus, seem to be more biologically appropriate for application in an inflamed acidic environment than ProRoot MTA, a very interesting finding for clinical situations involving, for example, root resorption or apicoectomy [31]. 

On the other hand, the term bioactivity is also related to the cellular effects induced by the release of biologically active substances and ions from the biomaterial [1]. For this reason, we analyzed the behavior of hPDLSCs in terms of apoptosis/necrosis, cell migration, and cell adherence in the presence of Bio-C Repair and Bio-C Sealer, three of the key processes that are critical to both angiogenesis and wound healing [32]. Our findings are consistent with previous observations that pointed to the capacity of cell migration in the presence of bioactive cements [33]. In our case, Bio-C Repair and ¼ Bio-C Sealer stimulated hPDLSCs to migrate. Correlating the cell-cytotoxicity assay with the cell-migration data, the lower migration recorded for cells exposed to undiluted and ½ Bio-C Sealer could be explained by the differences in composition of the materials. It may also be possible that the proprietary additives in the liquid may play a role in the biocompatibility of these materials. 

It is well known that the nature of the initial interaction between cells and biomaterials can influence cell function and their ability to produce an osteoid matrix and is a good predictor of their biocompatibility [34]. SEM assay pointed to differences in the morphology of hPDLSCs on the surface of Bio-C Sealer and Bio-C Repair. Cells cultured on Bio-C Repair were well spread, with a predominant spindle shape, and well-evident cytoplasmatic prolongations, extending to the limits. However, in the Bio-C Sealer group, less elongated and spindle-shaped cells were found on the surface. 

Finally, the surface characteristics of the endodontic cements were correlated with their biological properties [25,35], assessing the components of the materials by energy dispersive spectroscopy. Both Bio-C Repair and Bio-C Sealer revealed substantial amounts of calcium (Ca) which is compatible with the manufacturer’s indications. The release of calcium hydroxide is important not only because it plays a role in cementoblastic differentiation and dentine bridge formation, but also because it is responsible for the antimicrobial activity of this type of bioceramic material [26]. Zr was also observed in both materials, especially in Bio-C Sealer. The presence of zirconium oxide rather than bismuth oxide in these materials as a radiopacifying agent would prevent the discoloration that occurs in contact with some endodontic irrigants [36]. There were also traces of aluminum in Bio-C Sealer, which would have come from the cement phase of the material [37]. The difference in cytotoxicity between the cements analyzed in this study may be due to the specific chemical composition as well as to the solubility of each individual cement, indicating that other components contained in Bio-C Sealer, like tungsten or iron oxide, may affect the cell vitality of hPDLSCs. However, a limitation of our study is the lack of previous reports evaluating the cytotoxicity of Bio-C Repair and Bio-C Sealer.

## 5. Conclusions

The eluates form Bio-C Repair displayed higher cell viability and migration rates than Bio-C Sealer. Bio-C Repair discs showed increased cells attachment and proliferation compared with Bio-C Sealer.

## Figures and Tables

**Table 1:** Tested materials.

Materials	Manufacturer	Composition	Lot Number
**BIO-C Sealer**	Angelus, Rua Waldir Landgraf, Barrio Lindóia, Londrina, Brasil.	Powder: calcium silicate (Ca_2_SiO_4_), calcium oxide (CaO), zirconium oxide (ZrO_2_), iron oxide, silicon dioxide (SiO_2_), and dispersing agent. Dispersion agent/solid ratio = 0.52	43,207
**BIO-C Repair**	Angelus, Rua Waldir Ladgraf, Barrio Lindóia, Londrina, Brasil.	Powder: calcium silicate (Ca_2_SiO_4_),calcium oxide (CaO), zirconium oxide (ZrO_2_), iron oxide, silicon dioxide (SiO_2_), and dispersing agent Dispersion agent/solid ratio = 0.46	43,210

**Table 2:** Assessment of pH and inductively coupled plasma-mass spectrometry (ICP-MS) of cement extracts.

			Concentration of Element (mg/L Solution)			
	pH		Silicon	Strontium	Calcium	Zirconium
Complete DMEM	7.61 ± 0.03	Milli-Q water	0 ± 0	0 ± 0	0 ± 0	0 ± 0
Bio-C Sealer	8.40 ± 0.05	Bio-C Sealer	42.01 ± 0.01	0.3 ± 0.04	63.87 ± 0.01	0.13 ± 0.01
Bio-C Repair	8.33 ± 0.02	Bio-C Repair	14.90 ± 0.1	0.52 ± 0.01	38.32 ± 0.02	0.12 ± 0.02
